# Unveiling the hidden pathologies: preoperative endoscopic findings in patients with obesity undergoing bariatric surgery

**DOI:** 10.1186/s12893-024-02502-3

**Published:** 2024-07-24

**Authors:** Peirong Tian, Jing Fu, Yang Liu, Mengyi Li, Jia Liu, Jingli Liu, Zhongtao Zhang, Peng Zhang

**Affiliations:** 1 Division of Metabolic and Bariatric Surgery, Department of General Surgery, Beijing Friendship Hospital, National Clinical Research Center for Digestive Diseases, Capital Medical University, 95 Yong-an Road, Xi-Cheng District, Beijing, 100050 China; 2 Department of Endocrinology, Beijing Chao-Yang Hospital, Capital Medical University, Beijing, China

**Keywords:** Endoscopy, Bariatric surgery, Esophagitis, Risk factors, Pathology

## Abstract

**Background:**

Obesity is closely associated with upper gastrointestinal disorders. The recommendations for routine preoperative esophagogastroduodenoscopy (EGD) before bariatric surgery remains a topic of debate. This study aimed to describe the pathological endoscopic findings in individuals qualified for bariatric surgery.

**Methods:**

Retrospective analysis was conducted on preoperative gastroscopy reports of patients who underwent bariatric surgery at our hospital between October 2022 and October 2023.

**Results:**

A total of 405 patients were included in the study. The two most prevalent endoscopic findings during EGD in this patient cohort were chronic superficial gastritis (326/405, 80.5%) and reflux esophagitis (82/405, 20.2%). Some patients exhibited two or more abnormalities. Patients with reflux esophagitis were older, had a higher proportion of men, higher BMI, higher rates of smoking and drinking compared to those without it (*P* = 0.033, *P* < 0.001, *P* = 0.003, *P* = 0.001, and *P* = 0.003, respectively). Morbid obesity (*P* = 0.037), smoking habits (*P* = 0.012), and *H. pylori* infection (*P* = 0.023) were significant risk factors for reflux esophagitis in male patients, while age (*P* = 0.007) was the sole risk factor in female patients. No statistically significant differences were observed in surgical procedures between LA-A and B groups (*P* = 0.382), but statistically significant differences were noted between the nondiabetic and diabetic groups (*P* < 0.001).

**Conclusions:**

Preoperative EGD can unveil a broad spectrum of pathologies in patients with obesity, suggesting the need for routine examination before bariatric surgery. The findings of this study can guide bariatric surgeons in developing tailored treatments and procedures, thus significantly enhancing prognosis. Gastroscopy should be performed routinely in Chinese patients planning to undergo bariatric surgery.

## Introduction

Obesity and its associated metabolic syndromes are experiencing a relentless global surge [1, 2]. Utilizing criteria tailored for Chinese populations, recent national surveys indicate that approximately half of adults and a fifth of children exhibit either overweight or obesity, positioning China as the nation with the highest prevalence of overweight and obesity globally [3]. Extensive research underscores obesity as an unequivocal risk factor for prevalent chronic diseases encompassing type 2 diabetes (T2D), nonalcoholic fatty liver disease, cardiovascular ailments, and cancer [4–7]. Over the past decade, bariatric surgery has emerged as the most enduring and efficacious remedy for obesity, yielding sustained weight loss and favorable correlations with metabolic disorders related to obesity [8–10].

The imperative driving the preoperative esophagogastroduodenoscopy (EGD) prior to bariatric surgery is the identification and management of upper gastrointestinal (UGI) anomalies that may trigger symptoms or complications in the postoperative phase. Such pathologies encompass, albeit are not limited to, inflammatory alterations, peptic ulcer disease (PUD), hiatal hernia (HH), gastroesophageal reflux disease (GERD), and premalignant or malignant lesions of the upper gastrointestinal tract. The significance of these EGD findings could also hinge on the particular bariatric procedure under consideration and whether the intended intervention involves gastric exclusion [11–13].

Nevertheless, EGD is an invasive procedure associated with both financial implications and health risks. Evidence reveals elevated complication risks for patients with obesity undergoing EGD due to the heightened incidence of sleep apnea, desaturation, and electrocardiographic anomalies [14, 15]. In light of these factors, the advisability of routine preoperative endoscopic evaluations before bariatric surgery remains contentious, underscored by divergent viewpoints from multiple international organizations. The European Association for Endoscopic Surgery (EAES) endorses either barium swallow or EGD prior to bariatric surgery [16]. The International Federation for the Surgery of Obesity and Metabolic Disorders (IFSO) aligns with the EAES in emphasizing the importance of preoperative upper GI evaluation, stating that all patients, regardless of symptoms, should be considered for EGD. This recommendation is driven by the high prevalence of unexpected findings and the potential impact of UGI pathology on bariatric surgery outcomes [17]. In contrast, American scientific bodies such as the American Association of Clinical Endocrinologists (AACE), the American Society for Gastrointestinal Endoscopy (ASGE), the Society of American Gastrointestinal and Endoscopic Surgeons (SAGES), and the American Society for Metabolic and Bariatric Surgery (ASMBS) advocate for a personalized approach to upper endoscopy prior to bariatric surgery [18–20]. The conflicting guidelines raise the fundamental question of whether EGD should be routinely performed prior to bariatric surgery. Notably, gastric cancer stands as a leading cause of global cancer mortality, with a particularly high incidence in Asian countries, including China [21, 22]. Consequently, preoperative upper endoscopy has become the prevailing choice in the majority of Chinese bariatric centers.

In this study, we present an overview of the prevalence of clinically significant lesions identified during routine preoperative endoscopy of patients with obesity eligible for bariatric surgeries in our hospital. Our secondary objective was to ascertain potential predictors of reflux esophagitis (RE) that could influence surgeons in their selection of surgical techniques.

## Materials and methods

Retrospective analysis was conducted on preoperative gastroscopy reports of patients who underwent bariatric surgery at our hospital between October 2022 and October 2023. Irrespective of symptom presence, a routine upper gastrointestinal endoscopy was conducted as part of the patients’ preoperative assessment. This preoperative endoscopy was carried out in an elective setting by experienced endoscopists at our ambulatory endoscopy center. Patients who underwent revision bariatric procedures were excluded from the analysis, as were those who had undergone endoscopy at other centers. We retrospectively reviewed the UGI pathologies identified through visual examination during EGD and extracted clinical data, encompassing patient demographics, weight, BMI, presence of T2D and dyslipidemia, *Helicobacter pylori* infection status, as well as intoxication history (nicotine and alcohol consumption), from the Greater China Metabolic and Bariatric Surgery Database (GC-MBD). This database represents a prospective repository encompassing data from the vast majority of accredited bariatric surgery centers across China. Remarkably, it stands as the largest bariatric-specific registry in China. As of December 2022, 88 centres from 23 provinces, autonomous regions and municipalities in China have participated in the system and reported their data. The cumulative data volume of the database has exceeded 20 000 cases [23]. Trial Registration: This study was registered with ClinicalTrials.gov (NCT03800160) on April 1, 2018 (https://clinicaltrials.gov/study/NCT03800160).

Statistical analyses were executed using SPSS software (version 25.0; IBM Corp., Armonk, NY, USA). To evaluate the normality of continuous data distribution, the Kolmogorov-Smirnov test was employed, and data were presented as means (standard deviations) for normal distributions or medians (interquartile ranges) for skewed distributions. Categorical data were presented as numbers (percentages). To compare clinical characteristics between two groups, Student’s t-test, the Mann–Whitney *U*-test, or the Chi-square test were applied, as appropriate. Multivariate logistic regression analyses were undertaken to discern the risk factors associated with obesity-related reflux esophagitis. Adjusted odds ratios (ORs) and corresponding 95% confidence intervals (CIs) were computed. The potential risk factors considered encompassed age, sex, BMI status, smoking habits, drinking habits, diabetes presence, *Helicobacter pylori* status, and dyslipidemia. Subgroup analyses were executed according to sex. A significance threshold of two-sided *p*-values < 0.05 was deemed statistically significant.

## Results

During the study period, a total of 405 patients (male/female, 82/323) underwent bariatric surgery at our institution. Within the entire study cohort, the median age was 34 years (interquartile range: 30–37 years), while the median BMI was 39.33 kg/m^2^ (interquartile range: 33.23–45.27 kg/m^2^). Among the patients, 31.3% (*N* = 126) were classified as morbidly obese (BMI ≥ 40 kg/m^2^). Smoking history was noted in 135 patients (33.3%), while 177 patients (43.7%) had a history of alcohol consumption. T2D was present in 148 patients (36.5%), dyslipidemia in 338 patients (83.5%), and 57 patients were infected with *Helicobacter pylori* (14.1%). The clinical attributes of these index patients are detailed in Table 1.

**Table 1:** Clinical characteristics of the entire patient cohort (*N* = 405)

Variables	Total Population (*N* = 405)
Sex(M/F)	82/323
Age(years)	32 (27, 37)
BMI(kg/m^2^)	36.27 (32.70, 41.95)
BMI ≥ 40 kg/m^2^(N, %)	126 (31.3%)
History of Smoking (N, %)	135 (33.3%)
History of Alcohol Consumption (N, %)	177 (43.7%)
Diabetes (N, %)	148 (36.5%)
Infected with *Helicobacter pylori* (N, %)	57 (14.1%)
Dyslipidemia (N, %)	338 (83.5%)

The most prevalent endoscopic findings during EGD in this patient cohort were chronic superficial gastritis (326/405, 80.5%) and RE (82/405, 20.2%). Notably, some patients exhibited two or more concurrent abnormalities. Among the 82 patients diagnosed with RE, 61 were classified as grade A, and 21 as grade B, with no patients falling under grade C or D based on the Los Angeles classification. The frequencies of upper gastrointestinal pathologies identified through EGD are summarized in Table 2.

**Table 2:** Summary of preoperative gastroscopy findings in all 405 patients, categorized by major diagnosis group

Diagnosis	Number of cases (percentage of total sample)
Reflux esophagitis (LA grade A/B/C/D)	61/21/0/0
Formation of ectopic gastric mucosa of the esophagus	7
Raised lesions under the mucosal layer of the esophagus	6
Dilated veins of the esophagus	1
Hiatal hernia	13
Carditis	2
Raised lesions at the cardia/Polyps of cardia	5
Chronic superficial gastritis	326
Chronic atrophic gastritis	17
Erosive gastritis	65
Bile reflux gastritis	12
Gastric xanthoma	2
Gastric ulcer (Active/Healing/Scarring stage)	3/5/1
Nodular gastritis	3
Gastric polyp (Yamada type I/II/III/IV)	6/9/0/0
Ectopic pancreas	1
Gastric raised lesions	5
Pyloritis	1
Gastric retention	3
Duodenitis	16
Duodenal ulcer	9
Formation of ectopic gastric mucosa in the duodenum	2
Raised lesions under the mucosal layer of the duodenum	4
Situs inversus viscerum	1

The esophageal pathologies listed below were assessed based on endoscopic pictures only: reflux esophagitis (grade A–D), esophageal varices, and hiatal hernia

In order to further explore the risk factors associated with RE, the patient population was divided into two groups based on the presence or absence of this condition. Patients with RE were observed to be older (median age 34 years, interquartile range 30–37 years; *P* = 0.033), exhibited a higher proportion of men (37.8% vs. 15.8%, *P* < 0.001), possessed a higher median BMI (39.33 kg/m^2^, interquartile range 33.23–45.27 kg/m^2^; *P* = 0.003), and displayed higher rates of smoking (48.8% vs. 29.4%, *P* = 0.001) and alcohol consumption (58.5% vs. 39.9%, *P* = 0.003) compared to those without RE. Notably, there were no significant differences in T2D status, *H. pylori* infection, and blood lipid profiles. Refer to Table 3 for a comprehensive overview of the data.

**Table 3:** Comparison of clinical characteristics between patients with and without Reflux Esophagitis

Variables	Obesity with RE	Obesity without RE	*P* value
	(*N* = 82)	(*N* = 323)	
Sex(M/F)	31/51	51/272	< 0.001*
Age(years)	34(30, 37)	32(26, 36)	0.033*
BMI(kg/m^2^)	39.33(33.23, 45.27)	35.64(32.32, 40.40)	0.003*
BMI ≥ 40 kg/m^2^(N, %)	39(47.6%)	87(26.9%)	< 0.001*
History of smoking (N, %)	40(48.8%)	95(29.4%)	0.001*
History of alcohol consumption (N, %)	48(58.5%)	129(39.9%)	0.003
Diabetes (N, %)	34 (41.5%)	114(35.3%)	0.300
Infected with *Helicobacter pylori* (N, %)	10(12.2%)	47(14.6%)	0.435
Dyslipidemia (N, %)	65(79.3%)	273(84.5%)	0.253

Abbreviations: RE: reflux esophagitis

Continuous data presented as median (interquartile range) were compared using the Mann-Whitney *U* test

Categorical data presented as n (%) were compared using the chi-square test

*P < 0.05

**Fig. 1:**
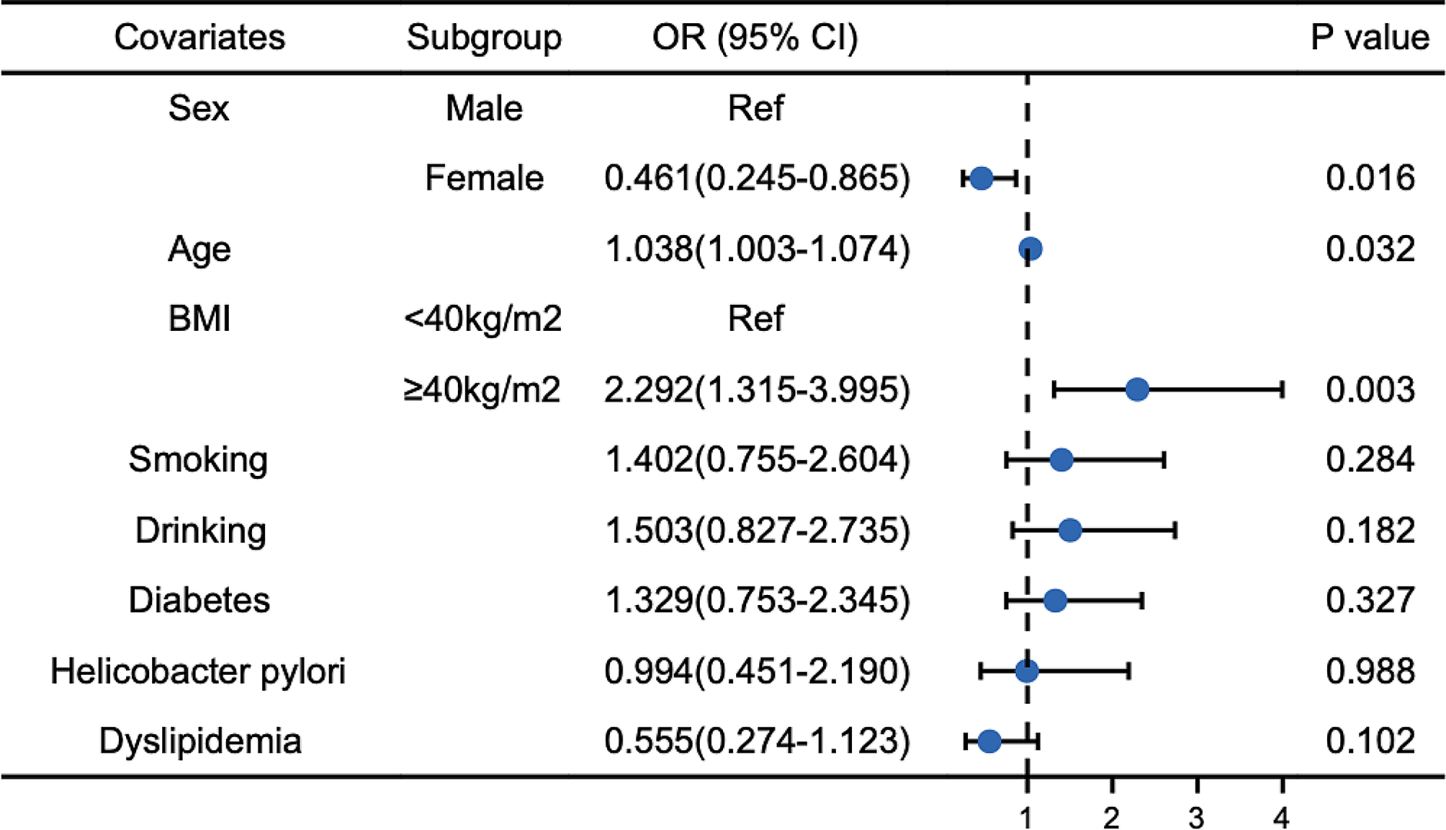
Multivariable logistic regression analysis for risk factors of reflux esophagitis. The figure illustrates the results of the multivariable logistic regression analysis conducted to identify the risk factors associated with reflux esophagitis (RE). The adjusted odds ratios (ORs) and corresponding 95% confidence intervals (CIs) are depicted for the various potential risk factors, including age, sex, BMI status, smoking status, drinking status, diabetes, *Helicobacter pylori* status, and dyslipidemia

**Fig. 2:**
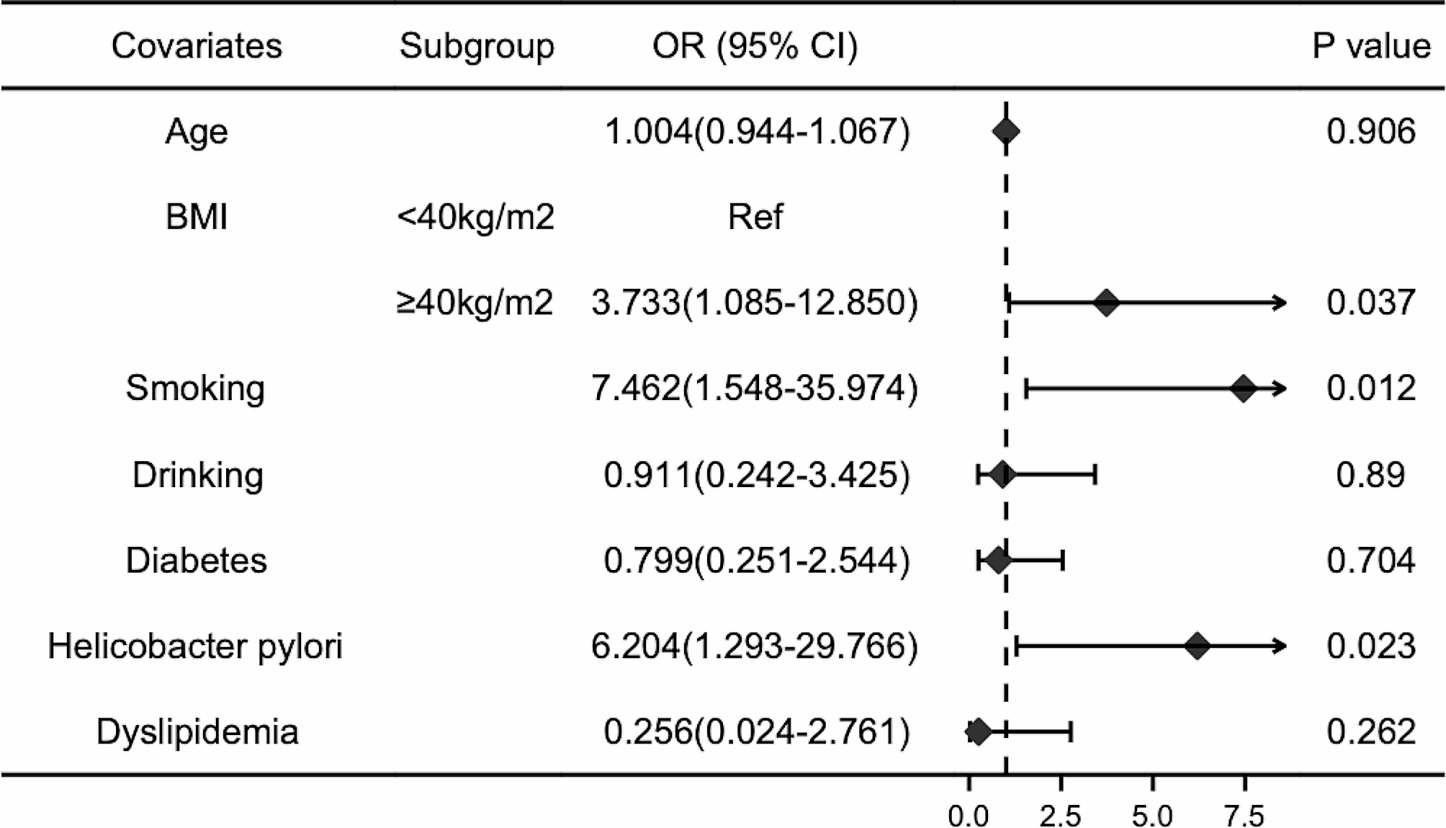
Risk factors of reflux esophagitis in male patients. This figure provides insights into the significant risk factors associated with reflux esophagitis (RE) among male patients. The multivariate logistic regression analysis highlights morbid obesity, smoking habits, and *H. pylori* infection as substantial risk factors contributing to the occurrence of RE in male patients

**Fig. 3:**
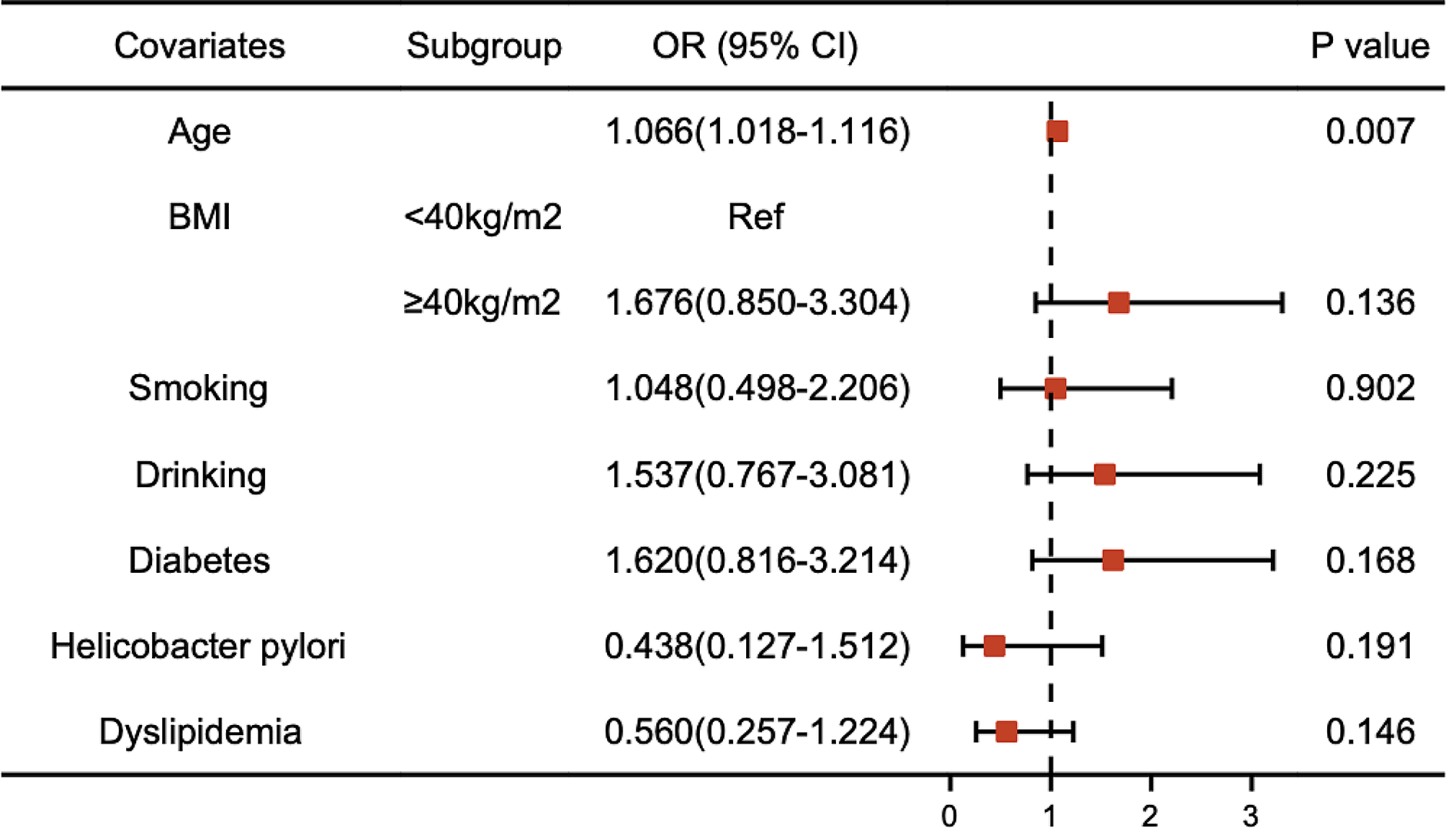
Risk factors of reflux esophagitis in female patients. The figure showcases the main risk factor linked to reflux esophagitis (RE) in female patients. The multivariate logistic regression analysis underscores age as the significant risk factor contributing to the development of RE in female patients

Subsequently, we conducted a multivariable logistic regression analysis utilizing eight indicators to assess potential risk factors for RE patients. The calculated ORs and corresponding 95% CIs are presented in Table 4; Fig. 1. In the final model, age (OR 1.038, 95% CI 1.003–1.074, *P* = 0.032) and morbid obesity (OR 2.292, 95% CI 1.315–3.995, *P* = 0.003) emerged as risk factors among patients with RE, while female sex exerted a protective role (OR 0.461, 95% CI 0.245–0.865, *P* = 0.016). Given the variations in smoking habits, alcohol consumption, and BMI between males and females, separate multivariate analyses were performed for each gender. The results revealed that morbid obesity (OR 3.733, 95% CI 1.085–12.850, *P* = 0.037), smoking habits (OR 7.462, 95% CI 1.548–35.974, *P* = 0.012), and *H. pylori* infection (OR 6.204, 95% CI 1.293–29.766, *P* = 0.023) were significant risk factors for RE in male patients (Table 5; Fig. 2), while age stood as the sole risk factor in female patients (OR 1.066, 95% CI 1.018–1.116, *P* = 0.007) (Table 6; Fig. 3).

**Table 4:** Multivariate logistic regression analysis of risk factors for patients with preoperative reflux esophagitis

Covariate	B	SE	Wald	*P*	OR	95% CI
Sex	-0.775	0.321	5.815	0.016*	0.461	0.245–0.865
Age	0.037	0.017	4.612	0.032*	1.038	1.003–1.074
BMI (≥ 40 kg/m^2^)	0.829	0.284	8.553	0.003*	2.292	1.315–3.995
History of smoking	0.338	0.316	1.148	0.284	1.402	0.755–2.604
History of alcohol consumption	0.408	0.305	1.784	0.182	1.503	0.827–2.735
Diabetes	0.284	0.290	0.962	0.327	1.329	0.753–2.345
Infected with *Helicobacter pylori*	-0.006	0.403	0.000	0.988	0.994	0.451–2.190
Dyslipidemia	-0.589	0.360	2.682	0.102	0.555	0.274–1.123

**Table 5:** Multivariate logistic regression analysis of risk factors for male patients with preoperative reflux esophagitis

Covariate	B	SE	Wald	*P*	OR	95% CI
Age	0.004	0.031	0.014	0.906	1.004	0.944–1.067
BMI (≥ 40 kg/m^2^)	1.317	0.631	4.364	0.037*	3.733	1.085–12.850
History of smoking	2.010	0.803	6.271	0.012*	7.462	1.548–35.974
History of alcohol consumption	-0.093	0.676	0.019	0.890	0.911	0.242–3.425
Diabetes	-0.225	0.591	0.145	0.704	0.799	0.251–2.544
Infected with *Helicobacter pylori*	1.825	0.800	5.203	0.023*	6.204	1.293–29.766
Dyslipidemia	-1.361	1.213	1.260	0.262	0.256	0.024–2.761

**Table 6:** Multivariate logistic regression analysis of risk factors for female patients with preoperative reflux esophagitis

Covariate	B	SE	Wald	*P*	OR	95% CI
Age	0.064	0.023	7.357	0.007*	1.066	1.018–1.116
BMI (≥ 40 kg/m^2^)	0.516	0.346	2.220	0.136	1.676	0.850–3.304
History of smoking	0.047	0.380	0.015	0.902	1.048	0.498–2.206
History of alcohol consumption	0.430	0.355	1.469	0.225	1.537	0.767–3.081
Diabetes	0.482	0.350	1.901	0.168	1.620	0.816–3.214
Infected with *Helicobacter pylori*	-0.827	0.633	1.706	0.191	0.438	0.127–1.512
Dyslipidemia	-0.579	0.399	2.109	0.146	0.560	0.257–1.224

Lastly, a focused analysis was undertaken among RE patients to assess the influence of reflux severity and diabetes status on the choice of surgical procedure. The distribution of surgical procedures and patient characteristics is outlined in Table 7. While no statistically significant differences were observed in surgical procedures between LA-A and B groups (*P* = 0.382), notable statistically significant differences were evident between the nondiabetic and diabetic groups (*P* < 0.001) (Table 7).

**Table 7:** Distribution of final surgical procedures for patients with preoperative reflux esophagitis

Patients with reflux esophagitis	SG	SG + HHR	RYGB	OAGB	Total	*P*
LA-A	39	4	15	3	61	0.382
LA-B	13	3	4	3	21	
Normoglycemic or pre-diabetic	40	6	3	1	50	<0.001*
Diabetes	11	1	16	4	32	

Abbreviations: SG: sleeve gastrectomy; SG + HHR: sleeve gastrectomy with hiatal hernia repair; RYGB: Roux-en-Y gastric bypass; OAGB: one anastomosis gastric bypass

*P < 0.05

## Discussion

Obesity and its association with various gastroesophageal pathologies have been widely documented in the literature [24]. Previous studies have highlighted an elevated occurrence of gastritis and ulcers in patients with obesity, leading to the proposal of a novel gastritis subtype termed “obesity-related gastritis” [25]. Furthermore, incidental findings of gastrointestinal tumors during bariatric surgery have been reported, underscoring the significance of pre-operative gastroscopy [26, 27]. These multiple concurrent abnormalities can group into three categories: (1) findings with minimal impact on medical or surgical management, such as mild mucosal inflammatory lesions or minor anatomical variations; (2) findings necessitating additional medical intervention, such as severe erosive gastritis/duodenitis, gastroduodenal ulcers, or *H. pylori* infection; (3) findings prompting alterations in surgical planning, including RE linked to HH and Barrett’s esophagus or precancerous/cancerous lesions. Based on these findings, surgeons might opt to delay, modify, or forego the intended procedure. However, in cases where screening is not conducted, surgeons must be prepared to address unforeseen findings in the operating room and engage in preoperative discussions regarding potential changes to the surgical plan, particularly in regions with high incidences of *Helicobacter pylori* infection and upper gastrointestinal malignancies, such as East Asia [28, 29].

Our study reinforces the established association between obesity and diverse gastroesophageal pathologies detectable through EGD. We observed a high prevalence of chronic superficial gastritis (80.5%), followed by reflux esophagitis (RE) (20.2%), hiatal hernia (HH) (3.2%), and peptic ulcers (4.4%). Notably, many patients exhibited multiple concurrent abnormalities, highlighting the complex interplay between obesity and gastrointestinal health. Our finding of 80.5% prevalence for chronic superficial gastritis aligns with prior research by Ng et al. who reported a similar prevalence (50%) in Asian bariatric surgery patients [30]. However, it is lower than the rates observed by Abd Ellatif et al. (23%) and Peromaa-Haavisto et al. (13.7%) in predominantly Arab and Caucasian populations, respectively [31, 32]. These discrepancies may be attributed to variations in study design, ethnicity, and diagnostic criteria. The prevalence of HH in our study (3.2%) is lower than that observed in other studies on bariatric surgery patients, which range from 16 to 52.5% [31, 33, 34]. This discrepancy may be due to differences in diagnostic techniques and the potential for smaller hernias to be missed during EGD.

Our RE prevalence of 20.2% is lower than that reported in several studies on bariatric surgery candidates, which range from 17 to 60% [31, 33, 34]. This difference might be due to our younger cohort, lower body mass index, and higher proportion of female patients compared to other studies. Notably, all RE cases in our study were grade A or B, suggesting a milder presentation compared to studies reporting higher prevalence rates. Our logistic regression analysis identified age, male sex, and obesity severity as independent risk factors for RE. This aligns with findings from previous studies by Colman et al. and Lee et al. who reported similar associations [22, 35]. Interestingly, subgroup analyses revealed that smoking and H. pylori infection were significant risk factors for RE in males but not females. This sex-based disparity aligns with observations made by Schigt et al. [36], suggesting potential gender-specific mechanisms underlying RE development in obese individuals. Further research is needed to explore these sex differences.

Presently, sleeve gastrectomy (SG) ranks among the most prevalent bariatric procedures globally due to its low complication rates, absence of gastrointestinal anastomosis, minimal malabsorption, patient acceptance, and adaptability to diverse bariatric procedures. However, the increasing adoption of SG has raised concerns about de novo or exacerbated GERD following the procedure [37]. During the International LSG Consensus Conference, 52.5% of general surgeons and 23.3% of bariatric surgeons regarded GERD as a contraindication to SG [38]. Given this perspective, severe reflux is considered a relative contraindication. Conversely, Roux-en-Y gastric bypass (RYGB) remains an optimal choice to prevent GERD symptom development and ameliorate preoperative reflux. Mechanistically, RYGB reduces acid production in the small gastric pouch and diminishes esophageal reflux due to the long Roux limb [39, 40]. Our analysis of surgical procedures based on preoperative gastroscopic findings showed that, interestingly, grade B patients did not exhibit a higher prevalence of RYGB relative to grade A patients. Notably, the majority of RE patients undergoing RYGB had T2D, likely attributed to the milder reflux degree (all grades A/B) and RYGB’s established status as a treatment for diabetes. Consequently, further in-depth studies are warranted for patients with grade C/D or Barrett’s esophagus to ascertain the influence of preoperative gastroscopic RE diagnosis on the choice of the final surgical approach.

Our study offers valuable insights into the association between obesity and EGD findings in bariatric surgery patients. However, limitations including its retrospective design, single-center recruitment, and gender imbalance necessitate further investigation. Future prospective multicenter studies with larger and more diverse cohorts are required to strengthen our findings and enhance generalizability. Additionally, standardization of endoscopy reporting and inter-endoscopist calibration would improve data accuracy. Investigating the cost-effectiveness of preoperative EGD and its impact on surgical decision-making and patient outcomes is also warranted. By addressing these limitations and pursuing these future research avenues, we can gain a more comprehensive understanding of the complex interplay between obesity, EGD findings, and patient outcomes in bariatric surgery. This knowledge can ultimately inform the development of evidence-based guidelines for the use of preoperative EGD in this patient population.

## Conclusions

The findings of this study highlight the potential of preoperative EGD to unveil a wide range of pathologies in individuals with obesity. Consequently, the consideration of routine EGD before embarking on bariatric interventions gains credence, particularly in light of its potential influence on therapeutic strategies. Notably, this recommendation holds significance for male patients characterized by older age, smoking history, and *Helicobacter pylori* infection.

## Data Availability

The datasets generated and/or analyzed during the current study are available from the corresponding author upon reasonable request.
